# Leveraging community engagement capacity to address COVID-19 disparities among Pacific Islander and Latinx Communities in Arkansas

**DOI:** 10.1017/cts.2020.562

**Published:** 2020-12-07

**Authors:** Pearl A. McElfish, Anna B. Cleek, Don E. Willis, Rachel S. Purvis, Laura P. James

**Affiliations:** 1 Internal Medicine, College of Medicine, University of Arkansas for Medical Sciences Northwest, Fayetteville, AR, USA; 2 Office of Community Health and Research, University of Arkansas for Medical Sciences Northwest, Fayetteville, AR, USA; 3 Department of Pediatrics, University of Arkansas for Medical Sciences, Little Rock, AR, USA

**Keywords:** COVID-19, disparities, community engagement, Pacific Islanders, Latinx

## Abstract

Northwest Arkansas, particularly Benton and Washington counties, is one of the highest COVID-19 hot spots in the United States (US), with more than half of all reported cases in this area identifying as Latinx or Pacific Islander, even though these communities account for less than 20% of the overall population. The University of Arkansas for Medical Sciences (UAMS) leveraged their existing relationship with 18 key community partners. Partners collaboratively developed a COVID-19 Response Strategy to ensure coordinated effort for Latinx and Pacific Islander communities with four interrelated strategies: health education, testing, contact tracing, and supported quarantine/case management.

## Introduction

The Centers for Disease Control and Prevention (CDC) documented the first United States (US) laboratory-confirmed case of COVID-19 on January 22, 2020 [1]. By June 2020, the US had over 3 million confirmed cases of COVID-19, and low-income neighborhoods showed high positivity rates for COVID-19 testing (30%–35%), compared to nationwide rates of 8.8% [2]. COVID-19 infections, hospitalizations, and deaths are disparate across racial and ethnic groups in the US, disproportionately impacting minority communities [3]. These disparities are magnified in certain areas of the US. For example, majority–minority counties in the US report infection rates 300% higher than that of White-majority counties, and death rates nearly 600% higher [4].

In June 2020, Northwest Arkansas, and in particular Benton and Washington counties, was one of the highest COVID-19 hot spots in the US. The racial and ethnic disparities of COVID-19 cases are so stark that the CDC conducted an in-depth community-level investigation in June and July 2020. The National Institutes of Health (NIH) followed suit in early August 2020. According to the CDC’s July 2020 report, 45% of all adult cases in Northwest Arkansas identified as Hispanic/Latinx, and 19% were Pacific Islander [5]. These communities only account for 17% and 2.4%, respectively, of the two-county population. Latinx and Pacific Islander community members encounter many socioeconomic challenges, including low educational attainment and unstable and dense housing. Latinx and Pacific Islander community members are often employed in low-wage jobs, primarily in the poultry industry, which are deemed essential and do not allow them to work from home [5,6].

For the past 5 years, the University of Arkansas for Medical Sciences (UAMS) has worked with Latinx and Pacific Islander community leaders and organizations to address health disparities and promote translational research. As the state’s only academic health center, we leverage a community-engaged approach to build trust between academic health centers and community stakeholders [7–12]. These partnerships are funded by the Clinical and Translational Sciences Award (CTSA) at UAMS and the CDC’s Racial and Ethnic Approaches to Community Health (REACH) program. These partnerships have long-standing community advisory boards and conducted several projects and research initiatives together. Details about the partnership and the collaborative work of the community–academic partnership are published elsewhere [13].

The mission of the CTSA at UAMS is to develop new knowledge and novel approaches that will measurably address the complex health challenges of rural and underrepresented populations. Grant resources provide training and support for community-based participatory research, community engagement, plain language communications, and the expansion of research in special populations [14,15].

The REACH program has a three-pronged focus for advancing REACH: supporting culturally tailored interventions to address preventable health conditions; linking community and clinical efforts to increase access to health care and preventive care programs at the community level; and the implementation, evaluation, and dissemination of practice- and evidence-based strategies to reduce health disparities in chronic conditions [16].

In early 2020, community-engaged partnerships utilized their collaborative capacity to address COVID-19 disparities in the Latinx and Pacific Islander communities. No CDC or NIH funding was spent on COVID-19-specific activities outlined in this article. However, partners continue to leverage existing community-based capacity to engage the Pacific Islander and Latinx community while developing and executing a COVID-19 Response Strategy to Reduce Health Disparities. Funding was provided through the Arkansas Department of Health’s distribution of CARES act funding [17]. This strategy is described below.

### A COVID-19 Strategy to Reduce Health Disparities for the Pacific Islander and Latinx communities in Northwest Arkansas

Since March 2020, 18 key community partners have had weekly meetings, and there is often daily communication between partners. Partners collaboratively developed a COVID-19 Response Strategy to ensure coordinated effort for Latinx and Pacific Islander communities with four interrelated strategies: health education, testing, contact tracing, and supported quarantine/case management. Partners are listed in Table 1.

**Table 1.** Community partners and the sector partners represent

Partner	Sector
Arkansas Children’s Hospital Northwest	Children’s hospital
Arkansas Coalition of Marshallese (ACOM)	Marshallese-focused nonprofit
Arkansas Department of Health	State Health Department
Arkansas Immigrant Defense	Immigrant-focused nonprofit
Arkansas United Community Coalition	Diversity-focused nonprofit
Community Clinic of Northwest Arkansas	Federally Qualified Health Center
Faith in Action Research and Resources Alliance	Community nonprofit
Hispanic Women’s Organization	Latinx-focused nonprofit
Kili Bikini Ejit	Marshallese-focused nonprofit
Marshallese Educational Initiative (MEI)	Marshallese-focused nonprofit
Mercy Health	Three-hundred and fifty-five-bed nonprofit hospital
Northwest Arkansas Council	Economic development organization
Northwest Health	For-profit health system with five hospitals in region
Republic of the Marshall Islands Consulate	Consulate office in Washington County Arkansas
Rooted NWA	Latinx-focused nonprofit
UAMS	Academic Medical Center
Veterans Administration of the Ozarks	Veterans health services in region
Washington Regional Medical Center	Four-hundred and twenty-five-bed nonprofit hospital

#### Strategy 1: Health education and prevention

Based on the recommendations from the CDC’s July 2020 report, partners co-developed a communications strategy designed specifically for COVID-19. This strategy covers four areas: prevention, testing, quarantine, and follow-up care. Prevention communication focuses on behaviors and practices individuals can take to reduce their risk of infection, including when and how to wear a mask, hand washing, and how to stay connected with family and friends while maintaining an appropriate physical distance.

Communications about COVID-19 testing focuses on describing when and where people can be tested, provides bilingual videos for those places doing self-administered testing swabs, provides information about costs, and informs on safe behaviors while waiting for test results. The quarantine and isolation guidance focuses on criteria for when quarantine and self-isolation are appropriate and explains the available social services to support those efforts. Follow-up care covers symptom management and when and how to seek additional care if needed. Partners also developed targeted communications for high-risk community members, specifically addressing pregnancy, diabetes, mental health, and asthma. Small business and faith-based tool kits were created and have been distributed to more than 120 local small businesses within the Latinx and Pacific Islander communities. Communications primarily focus on nontraditional and unpaid media.

All communications are in English, Spanish, and Marshallese (the native language of most Pacific Islanders in Northwest Arkansas) and leverage local Latinx and Pacific Islander community leaders. The communications materials can be found at https://northwestcampus.uams.edu/ochrcovid/. An outline of communications tools developed as of November 30 is listed in Table 2. New communication tools are added frequently.

**Table 2.** Types and estimated reach of COVID-19 communications materials

Communications medium	Number and estimated reach
Facebook live sessions	Twenty-nine videos developed and disseminated via Facebook with an estimated average reach of 21,000 each video
YouTube videos	Twenty-one videos developed and distributed with an average reach of 7,120
Printed materials	Forty-three printed educational documents developed and distributed to more than 10,000 people via food boxes, nonprofit organizations, and testing centers
Radio interviews	Nine radio interviews with an estimated reach of 12,000
Faith-based tool kit	One tool kit in each of the 3 languages with an estimated reach of 50 churches and 40,000 community members
Small business tool kit	One tool kit in each of the 3 languages with an estimated reach of 120 small businesses and 150,000 community members

#### Strategy 2: Testing

The COVID-19 Strategy to Reduce Health Disparities for the Pacific Islander and Latinx communities is led by the Federally Qualified Health Center (FQHC), and employs the resources of UAMS, Arkansas Department of Health offices, and local healthcare providers for testing. Based on the CDC July 2020 report, testing primarily focuses on organizations and/or neighborhoods with a high number of cases so that partners can quickly target hot spots and reduce the spread. Targeted serial testing ensures rapid testing of all contacts of known or suspected COVID-19 cases. When a positive case is identified, all of their contacts are offered in-home testing. If the contact(s) prefer to not have testing at their home, they are directed to drive-through testing centers. For in-home testing, a nurse-led team of healthcare workers provide testing to contacts and all household members without contacts leaving their home. In addition, drive-through testing centers are distributed throughout two counties at local healthcare organizations and mobile testing teams conduct tests at churches, worksites, and housing complexes. Most importantly, all testing locations have bilingual staff. Community partners play an important role in helping identify the community-based location for mobile testing and encouraging community members to get tested at any of the testing locations.

#### Strategy 3: A dedicated contact tracing center with bilingual workers

The COVID-19 Strategy to Reduce Health Disparities for the Pacific Islander and Latinx communities focuses on establishing a bilingual contact tracing center in the region that fully integrates with the Arkansas Department of Health. The contact tracing center is staffed with bilingual contact tracing staff. The bilingual contact tracing center utilizes the same software, policies, and procedures as the Arkansas Department of Health and includes a designated process of identification and follow-up of all persons who may have come into contact with a person infected with COVID-19.

Since a positive test for COVID-19 is a mandatory reportable diagnosis to the Arkansas Department of Health, any new cases are reported, and those whose preferred language is Marshallese or Spanish are referred to the bilingual contact tracing center. Bilingual staff at the contact tracing center reach out to the case and determine direct contacts for the case during the period from 2 days before symptoms started until the case quarantined. The case is asked to inform all contacts that someone from the contact tracing center will be calling them. After the case alerts those contacts to expect a call, contact tracing staff will contact them to begin the screening process and advise quarantine. This quarantine applies to their households as well as any other direct contacts. This initial survey triggers a 14-day tracking period, which consists of daily communication with each contact under quarantine. All contact tracing data are entered into the Arkansas Department of Health software and uploaded to the department at the end of each day. All cases and contacts receive education about how to isolate (Strategy 1). Contacts are offered testing (Strategy 2). All cases and contacts who need assistance with food, housing, and medication work with a social worker and bilingual navigators to identify resources to meet those needs (Strategy 4).

#### Strategy 4: Enhanced case management and supported quarantine

A COVID-19 diagnosis often exacerbates the socioeconomic challenges that the Pacific Islander and Latinx populations face in Northwest Arkansas. Bilingual social workers, nurses, and community health navigators make up the enhanced case management team. It is critical to provide support services for both cases and contacts as they self-quarantine. Needed support includes essentials like food and medications, coordination with worksites, and coordination with community behavioral health services. Standard contact tracing encourages contacts to stay home and maintain distance from others until 14 days after their last exposure. The enhanced case management process elevates this with follow-up communication with the person who has COVID-19 and contacts to follow all quarantine guidelines. The enhanced case management also discusses their health and inquires about new symptoms. The staff provides resources, education, information, and connection with health care and community-based support organizations. They arrange for both food deliveries and prescription drug refills, if needed, to facilitate the contacts’ ability to remain at home. The nonprofit organizations listed in Table 1 have funding and actively take part in ensuring cases and contacts have the support they need.

## Conclusion

The COVID-19 Strategy to Reduce Health Disparities for the Pacific Islander and Latinx communities in Northwest Arkansas demonstrates how community-based participatory research and programmatic networks funded by NIH and the CDC can be quickly leveraged to address COVID-19, which has disproportionately affected Latinx and Pacific Islander community members. This example shows the importance of building and sustaining community-based research and programmatic networks. The regional collaborative in Northwest Arkansas plans to leverage their network in the future to ensure Pacific Islander and Latinx communities have the opportunity to participate in vaccine studies and the distribution of COVID-19 vaccines.
